# Initial Respiratory System Involvement in Juvenile Idiopathic Arthritis with Systemic Onset Is a Marker of Interstitial Lung Disease: The Results of Retrospective Cohort Study Analysis

**DOI:** 10.3390/jcm13133843

**Published:** 2024-06-29

**Authors:** Konstantin E. Belozerov, Eugenia A. Isupova, Natalia M. Solomatina, Ekaterina V. Gaidar, Maria A. Kaneva, Irina A. Chikova, Olga Kalashnikova, Alla A. Kuznetsova, Dmitry O. Ivanov, Mikhail M. Kostik

**Affiliations:** Hospital Pediatrics, Saint-Petersburg State Pediatric Medical University, 194100 Saint Petersburg, Russia; biancolago@bk.ru (K.E.B.); tatasolo@icloud.com (N.M.S.); koira7@yandex.ru (O.K.); pediatr-kuznetsova@yandex.ru (A.A.K.); spb@gpmu.org (D.O.I.)

**Keywords:** SJIA, LD-SJIA, interstitial lung disease, pleuritis, arthritis, lungs, anakinra, canakinumab, tocilizumab

## Abstract

**Background:** Pulmonary involvement in systemic juvenile idiopathic arthritis (SJIA) is a rare but dangerous complication. The main risk factors are already known, such as macrophage activation syndrome, a refractory course of systemic juvenile arthritis, infusion reaction to interleukin 1 and/or interleukin 6 blockers, trisomy 21, and eosinophilia. However, information about respiratory system involvement (RSI) at the onset of SJIA is scarce. Our study aimed to evaluate the specific features of children with SJIA with RSI and their outcomes. **Methods:** In a single-center retrospective cohort study, we compared the information from the medical records of 200 children with SJIA according to ILAR criteria or SJIA-like disease (probable/possible SJIA) with and without signs of RSI (dyspnea, shortness of breath, pleurisy, acute respiratory distress syndrome, and interstitial lung disease (ILD)) at the disease onset and evaluated their outcomes (remission, development of chronic ILD, clubbing, and pulmonary arterial hypertension). **Results:** A quarter (25%) of the SJIA patients had signs of the RSI at onset and they more often had rash; hepato- and splenomegaly; heart (pericarditis, myocarditis), central nervous system, and kidney involvement; hemorrhagic syndrome; macrophage activation syndrome (MAS, 44.4% vs. 9.0%, *p* = 0.0000001); and, rarely, arthritis with fewer active joints, compared to patients without RSI. Five patients (10% from the group having RSI at the onset of SJIA and 2.5% from the whole SJIA cohort) developed fibrosing ILD. All of them had a severe relapsed/chronic course of MAS; 80% of them had a tocilizumab infusion reaction and further switched to canakinumab. Unfortunately, one patient with Down’s syndrome had gone. **Conclusion:** Patients with any signs of RSI at the onset of the SJIA are required to be closely monitored due to the high risk of the following fibrosing ILD development. They required prompt control of MAS, monitoring eosinophilia, and routine checks of night oxygen saturation for the prevention/early detection of chronic ILD.

## 1. Introduction

Systemic juvenile idiopathic arthritis (SJIA) is a rare, and the most dangerous, juvenile idiopathic arthritis (JIA) category, associated with joints, internal organ involvement, and macrophage activation syndrome (MAS) [1].

Chronic interstitial lung disease (ILD) is a relatively newly discovered life-threatening complication of sJIA, with a rise in frequency in the last decades. ILD in SJIA patients might be presented with pulmonary arterial hypertension (PAH) or interstitial lung disease, lipoid pneumonia (LP) and pulmonary alveolar proteinosis (PAP), associated with disrupted clearance and storage of lipids or surfactant, respectively. In some patients, PAH and ILD can coexist [2,3,4]. The number of patients with SJIA and ILD has risen since the beginning of the current century, which is correlated with raised implementation of biologic drugs for the treatment of SJIA [3].

Several studies have identified the predictors of chronic lung involvement in SJIA: early onset (usually less than two years); predominant systemic features; severe, relapsed, or chronic MAS; eosinophilia; progressive lymphopenia; atypical itchy rash; hypersensitive reaction on biologic agents against interleukin 1 (IL1) or IL6 (anaphylaxis); and trisomy 21 syndrome [2,3,5].

Hyper-activation of the γ-interferon (IFNγ) pathway with increased production of interleukine-18 are key pathogenic points of this disease [2,3]. Treatment with emapalumab (anti-γ-interferon) and interleukine-18 binding protein look like promising options, but the results are still limited [5].

Pulmonary involvement occurs in 5% of adult-onset Still disease (AOSD), similar to SJIA, but the information is also scarce [6,7]. Among the signs of respiratory system involvement (RSI) at the onset of the SJIA, pediatric rheumatologists are familiar with pleuritis, which is a part of the SJIA classification criteria, but information about other symptoms is limited too [8,9].

Our study aimed to evaluate the specific features of children with SJIA with RSI and their outcomes.

## 2. Methods

In a single-center retrospective cohort study, the information from the medical records of children who had the following *inclusion criteria* was included:

1. Patients must have a diagnosis of SJIA according to International League Against Rheumatism (ILAR) criteria or SJIA-like disease (probable/possible SJIA) and meet the recently proposed criteria for SJIA [9].
2. The age of SJIA onset is less than 18 years.

In every patient, we evaluated the initial following parameters at the onset of SJIA:

- Demography: sex, onset age, treatment before the established diagnosis of SJIA, correspondence to the Yamaguchi and Fautrel criteria for adult-onset Still disease [10,11].
- All routine clinical and laboratory features of SJIA.
- Presence of MAS according to the Ravelli A et al. criteria from 2005 and EULAR/ACR/PRINTO 2016 versions, criteria for hemophagocytic lymphohistiocytosis (HLH2004), and HScore calculation [12,13,14,15].
- Signs of RSI, such as dyspnea and shortness of breath, associated with other signs of RSI, such as pleurisy, local auscultation phenomenon (crackles, crepitations), acute respiratory distress syndrome (RDR), clubbing, ILD, and PAH.
- Data from chest high-resolution computed tomography (HRCT). According to the national recommendations, a chest HRCT is a mandatory examination in all children with suspected SJIA [16,17].
- Treatment: corticosteroids, biologic (bDMARDS), and non-biologic disease-modifying anti-rheumatic drugs (nbDMARDS).
- Outcomes: remission, improvement or active disease, chronic ILD development.

We compared sJIA patients with and without signs of respiratory involvement at the disease onset and evaluated the following treatment and outcomes in both groups.

**Ethics:** Written consent to participate in this study was obtained following the Declaration of Helsinki. Written informed consent was provided by the participant’s legal guardian/next of kin. Consents to the use of patients’ data in an anonymous form are available in the patient’s case histories. The data have been properly anonymized. The ethics committee of St. Petersburg State Pediatric Medical University approved the protocol for this study (No. 18/01; 27 October 2022).

### Statistical Analysis

The sample size was not calculated initially. Each quantitative variable was tested using the Kolmogorov–Smirnov test to ensure the normality of distribution. No normal distribution was found. Descriptive statistics were presented as median (Me) and quartiles (25%; 75%) for quantitative variables, absolute value, and proportion (%) for categorical variables. The Mann–Whitney U test was used to compare quantitative variables in two independent groups, and the chi-square test was used to compare categorical variables, or Fisher’s exact test if the number of expected observations in any of the cells of the four-field table was less than five. For quantitative variables, cut-off values were calculated using AUC-ROC analysis (AUC—area under the curve) with a 95% confidence interval (CI). The odds ratio (OR) calculation, independent of the time of events development, was performed using a 2 × 2 ratio (OR) calculation with a 95% confidence interval (95% CI) grouping based on binary characteristics tables. Sensitivity (Se) and specificity (Sp) were assessed for each parameter. The analysis of sensitivity and specificity with odds was performed to find the predictors of involvement of the respiratory system. Differences or relatedness were considered statistically significant at *p* < 0.05.

The construction of a prognostic model of the risk of a certain outcome was carried out using the multivariate logistic regression method. The selection of independent variables was carried out using the method of step-by-step direct selection using Wald statistics as an exclusion criterion. The statistical significance of the resulting model was determined using the χ^2^ test. The measure of certainty, indicating the portion of variance that can be explained using logistic regression, was used in our study as the Nigelkirk index. Differences were considered statistically significant at *p* < 0.05. Statistica software (release 10.0, StatSoft Corporation, Tulsa, OK, USA) and MedCalc (MedCalc Software, Belgium) were used for data analysis.

## 3. Results

### 3.1. Patients’ Demography

Our cohort of sJIA patients was characterized by a slight predominance of girls (59.0%) with preschool onset age—5.3 (3.0; 9.1) years. The median systemic score was 2.0 (2.0; 3.0), and the main clinical features were as follows: rash, 76.5%; arthritis, 54.5%; arthralgia without arthritis, 10.0%; hepatomegaly, 65.5%; lymphadenopathy, 61.5%; splenomegaly, 46.0%; heart, 24.0%; CNS, 14.5%; and kidney, 10.5% involvement. The characteristics of our cohort are in Table 1. At the onset of sJIA, 50 (25%) of the patients had different types of respiratory involvement.

### 3.2. Clinical Features

The main respiratory symptoms were as follows: dyspnea, shortness of breath, pleurisy, acute RDR, and ILD. The features of chronic lung involvement—clubbing, PAH—were not seen at onset and were observed only in patients with chronic ILD at least one to two years after onset. Patients with initial respiratory involvement had higher systemic scores and more often had the features of rash, hepato- and splenomegaly, heart (pericarditis, myocarditis), central nervous system, kidney involvement, and hemorrhagic syndrome compared to patients without respiratory involvement. Patients with respiratory involvement had arthritis rarely and with a fewer number of active joints. The data are in Table 1.

### 3.3. Laboratory Findings

Patients with respiratory involvement had lower levels of hemoglobin, platelets, total protein, albumin, fibrinogen, and prothrombin and higher levels of alanine transaminase (ALT), aspartate transaminase (AST), γ-glutamyltransferase (GGT), lactate dehydrogenase (LDH), ferritin, and triglycerides compared to children without respiratory involvement. The data are in Table 1.

### 3.4. Macrophage Activation Syndrome Assessment

Since patients had clinical and laboratory features that were typical for MAS, the diagnostics of this condition was performed with different tools. Patients with respiratory involvement had higher levels of HScore and a higher risk of hemophagocytosis according to this diagnostic set of criteria. The positivity of MAS ranged from 37.8 to 44.4% according to the present diagnostic sets (HLH2004: 37.8%, MAS 2005: 40.0%, and MAS2016: 44.4%). In patients without initial respiratory involvement, the frequency of MAS was significantly lower and ranged from 3.5 to 9.0% according to the abovementioned criteria. The data are in Table 1.

### 3.5. Predictors of the Respiratory System Involvement

In the univariate analysis, the following predictors with the highest sensitivity, specificity, and odds ratio were associated with RSI at onset of the SJIA: ICU admission, hemorrhage, heart involvement, hypoalbuminemia (≤26.0 g/L), thrombocytopenia (≤211.0 × 10^9^/L), hypertriglyceridemia (>2.0 mmol/L), systemic score > 3.0, transaminitis (AST > 90.0 U/L). The complete list of the predictors is in Table 2.

Multivariate analysis found the main predictors of RSI in SJIA patients were as follows: hypoalbuminemia (≤26.0 g/L), systemic count > 3.0, and heart involvement (R^2^ = 0.62, *p* = 0.000001).

### 3.6. Outcomes

Interestingly, we did not observe significant differences in the main disease outcomes in children with and without initial RSI in terms of remission, treatment options, and efficacy. The main outcomes during the last observation are in Table 3.

The frequency of TCZ infusion reaction was higher in the RSI group (OR = 4.1 (0.7; 25.4), sensitivity: 7.7%, specificity: 98.0%, *p* = 0.106). The remission rate was lower in the RSI group (OR = 2.1 (0.9; 4.8), sensitivity: 22.0%, specificity: 88.0%, *p* = 0.082). All patients with tocilizumab infusion reactions were switched onto canakinumab.

Five patients (10%) from the group having RSI at the onset of SJIA further developed fibrosing interstitial lung involvement. All of them (5/5) had a severe relapsed/chronic course of MAS; 80% of them had a tocilizumab infusion reaction (*n* = 4/5) and required the switching of the biologics. In most cases (4/5) tocilizumab was switched to canakinumab. Unfortunately, one patient with Down’s syndrome had gone. None of the patients who did not have involvement of the respiratory system at the onset developed subsequent chronic fibrosing interstitial lung involvement. So, initial respiratory involvement was the strongest predictor of following chronic LD-SJIA.

## 4. Discussion

### 4.1. A Brief Report of Our Results

At the SJIA onset, 25% of patients had different types of respiratory involvement, and, in the majority of the cases, it was associated with active MAS. The main predictors of RSI were systemic score > 3, heart involvement, and low albumin level. Initial RSI led to fibrosing ILD development in the future in 10% of the sJIA patients.

### 4.2. Epidemiology and Clinical Features

SJIA is a variant of an autoinflammatory disease that is characterized by fever, rash, arthralgia/arthritis, and multiorgan involvement [1,18]. According to national epidemiological studies, the frequency of systemic arthritis is very different and ranges from 5% in Africa up to 40% in Japan among whole JIA patients [19,20]. At the moment, in 2024, there were 2081 children and adults with SJIA in our country according to official sources, which is approximately 7.5% of the whole JIA cohort [16].

LD-SJIA is a new challenging complication in the spectrum of clinical manifestations of SJIA. The number of cases has grown in the last two decades. The first case series was published by Kimura Y et al. in 2013 and contained information about 25 patients [3,4]. Now, we have information about 14 patients with LD-SJIA in our country, which is nearly 0.67% of the patients with SJIA (personal communications), but the frequency of LD-SJIA in our cohort is higher at 2.5%. A quarter of our SJIA cohort had respiratory involvement at the onset of the disease, and 4% (5/200) developed chronic fibrosing forms. The prognosis for LD-SJIA patients has remained very grave but has become better. In the first 25 cases published by Y. Kimuro et al. (2013), 68% of patients whose lung involvement was diagnosed died in 8.8 ± 11.4 months from the pulmonary manifestation [4]. Further studies showed better outcomes with mortality near 5%, which is much more than in patients with SJIA without lung damage [21]. In our cohort, one patient (0.5%) died, which is 2% from patients with initial RSI and 10% from those having fibrosing lung involvement.

Several risk factors of LD-SJIA have been previously described. They were the prevalence of systemic manifestations, presence of MAS, eosinophilia, atypical rash, lymphopenia, and trisomy 21 [2,3].

In our cohort focused on the initial respiratory involvement, some risk factors overlapped: macrophage activation syndrome and high systemic score were presented in our patients. The main clinical distinguishing features associated with RSI were more frequent rash, hepatosplenomegaly, heart, CNS, renal involvement, coagulopathy, systemic score, and a lower number of active joints. Patients were more systemic than articular. The frequency of tocilizumab anaphylaxis was higher in our group, but it was not an early symptom, because tocilizumab was prescribed in patients with a confirmed diagnosis of SJIA, and anaphylaxis was noted on the second or third tocilizumab infusion, which was at least 2–4 weeks after the diagnosis of SJIA. In common, patients with chronic ILD were similar from the clinical point of view to patients with ILD, as described earlier [2,3,4,21].

### 4.3. Trisomy 21

In our group, trisomy 21 with eosinophilia was very rare. A previous study mentioned that trisomy 21 syndrome increased the frequency of ILD up to 50 times in SJIA [2]. Trisomy 21 syndrome might be associated with different lung involvement due to immune dysregulation [22,23]. There are data suggesting interferon up-regulation in trisomy 21 syndrome, which closely resembles monogenic interferonopathy; there, ILD is typical, e.g., in SAVI-syndrome [23,24]. Also, many patients with trisomy 21 frequently have congenital heart disease and undergo cardiac surgery. All the abovementioned factors may interact in the pathogenesis of lung involvement [25]. In such cases, it is difficult to position the role of SJIA in lung involvement in patients with trisomy 21 syndrome. In the literature, we found few clinical descriptions of combined trisomy 21 syndrome and SJIA. In our cohort, there was one female patient with trisomy 21, who had gone during MAS and LD-SJIA. Worley E et al. described a 21-month-old male toddler with trisomy 21 and SJIA who developed MAS. The treatment included prednisone and early prescribed IL-1 blocker-anakinra (without any anaphylaxis reactions). After ILD had occurred, tofacitinib was added and the patient showed improvement [26]. The efficacy of JAK inhibitors in such cases is similar to monogenic interferonopathies. JAK inhibitors indirectly suppress interferon hyperproduction, which is used in clinical practice.

### 4.4. Eosinophilia

Eosinophilia is another risk factor for LD-SJIA patients. Several recent studies suggested that LD-SJIA could have a similar pathogenesis and drug reaction to eosinophilia and systemic symptoms (DRESS) syndrome, where eosinophilia is a part of the immune reactions. HLA-DRB1*15 is assumed to be a factor for adverse drug reactions of bDMARD among patients with SJIA [27]. Wobma H et al. described 75 patients with SJIA and 41 patients with other JIA. Eosinophilia was observed during the treatment with IL-1/IL-6 inhibitors in SJIA and non-systemic JIA. MAS, preceding treatment with bDMARDs, was associated with a higher rate of subsequent eosinophilia on bDMARDs. The authors suggested eosinophilia could be associated with an aggressive disease course and LD-SJIA development. Also, there was a high frequency of DRESS (*n* = 45; 49%) in sJIA patients who were positive for HLA-DRB1*15 alleles [28]. Similar to children, the majority of the patients with AOSD developed DRESS-type reactions and were carriers of HLA-DRB1*15 haplotypes [29]. DRESS reactions included eosinophilia, the elevation of AST and ALT, and non-evanescent rash. So, eosinophilia as a part of hypersensitivity to IL1- or IL6-blockers is obvious, but possibly more information about HLA-DRB1*15 as the potential predictor of biological DMARDs anaphylaxy is needed [28]. We did not see eosinophilia at the disease onset as well as in the follow-up in our cohort.

Considering many cases of LD-SJIA developed in patients receiving IL1- or IL6-blockers, especially those having experienced switching between two classes of the drugs, hypersensitivity reactions to these drugs required the search for other approaches to the treatment of such patients. Macrophage activation syndrome, hyperproduction of IL-18, and interferon-γ are considered the main targets for therapy. It is known that patients who received regular therapy with an IL1β inhibitor (canakinumab) continued to have increased levels of IL18 in remission. This fact may explain the delayed development of ILD in patients undergoing biological therapy, especially in remission [30].

### 4.5. MAS, Subclinical MAS, and LD-SJIA

MAS, being the main threat of SJIA, belongs to the family of hemophagocytic syndromes occurring in rheumatic disease patients. MAS is characterized by the overproduction of inflammatory cytokines, particularly IL-1β, IL6, IL18, and IFNγ. The incidence of acute/evident MAS is about 10% (8–17%) in sJIA patients, but subclinical forms of the disease are assumed in approximately 35% of SJIA cases [2,3,4,31]. The term “subclinical MAS” is perspective but has not yet received its diagnostic criteria. It could be a useful term for the explanation and earlier detection of SJIA patients with a high risk of developing LD-SJIA. The term “subclinical MAS” is very controversial and there are no definite criteria [31]. A quantitative measure of MAS score looks like a promising tool for such cases; their dynamic is very important. This tool is required to be validated for rheumatic disease patients. Recently, remission criteria for MAS were proposed, which include resolution of MAS-related clinical signs and symptoms AND normalization of laboratory parameters relevant to MAS: WBC > lower limit of normal (LLN), platelet count > LLN, LDH < 1.5 upper limit of normal (ULN), ALT < 1.5 ULN, AST < 1.5 ULN, fibrinogen > 100 mg/dL, and ferritin ≤ 80% from values at screening or baseline (whichever is higher) or <2000 ng/mL, whichever is lower [32].

It is necessary to note that these criteria are more convenient for assessment of the efficacy of the treatment of MAS than interfering with a complete resolution of the MAS. Some patients who met these criteria overlap with subclinical MAS and could be at risk of a further MAS flare with major organ involvement (lung, liver).

Duration of ICU stays and hospital admissions, duration of fever, ventilation support requirement, death, and reduction of several hospitalizations long-term were proposed as possible outcome measures for refractory MAS [32].

Several studies showed the association between MAS and pulmonary and kidney involvement [33,34]. Children with sJIA and MAS who developed thrombotic microangiopathy had lung involvement in 47.1% of cases. In our group, there were no cases of TMA, and kidney involvement was limited to transient proteinuria (less than 1.0 g/24 h).

### 4.6. Diagnostics of ILD

In adult rheumatoid arthritis and AOSD, the lung ultrasound with the assessment of B-lines and serum Krebs von den Lungen-6 antigen (KL-6) might be applied for the early detection of ILD [35]. KL-6 antigen is expressed after alveolar cell type II damage, so it elevates in ILD [36]. KL-6 antigen could be a useful biomarker of ILD in pediatric rheumatic disease but nonspecific to concert nosology (SJIA, systemic lupus erythematosus, dermatomyositis, etc) [37,38]. HRCT is the most common tool for diagnostics of ILD in SJIA. The typical changes characteristic of ILD are pleural and septal thickening, bronchial wall or peribronchovascular thickening, “tree-in-bud” opacities, “ground-glass” opacities, peripheral consolidation, and lymphadenopathy [3].

### 4.7. Treatment

Treatment approaches include new drugs such as the interferon-gamma inhibitor emapalumab, effective in the treatment of familial hemophagocytic lymphohistiocytosis, and interleukin-18 binding protein, effective in NLRC4-periodic syndrome, where relapsed MAS is a main part of the disease [5,39]. The use of IL18-binding protein to inhibit this cytokine is being proposed, as other available targeted therapies have failed [40,41].

Inhibitors of JAK are expected to be a promising tool for the treatment of ILD [5,42,43]. Nintedanib, an intracellular tyrosine kinase inhibitor, is approved for progressive fibrosing interstitial pulmonary disease, particularly applied in systemic scleroderma [44,45].

### 4.8. Limitations

The main limitations were related to the retrospective manner of this study with some missing data. The absence of a unique treatment protocol, different access to the biologics, and different times before biologics during the years might lead to different outcomes. The impossibility of seeing patients after 18 years, different observation periods, and relatively low observation period might influence this study’s results.

## 5. Conclusions

The presence of MAS, low albumin level, high number of systemic features, and cardiovascular system involvement are associated with RSI in patients with SJIA. Respiratory system involvement at the onset, along with known clinical and laboratory predictors of chronic pulmonary involvement, makes it possible to identify patients with a risk of developing primary chronic or delayed pulmonary disease. Careful monitoring of SJIA activity and control of active and subclinical MAS is required for personalized therapy. Diagnosis and treatment of children with ILD in SJIA require a multidisciplinary approach with the participation of a rheumatologist, chest physician, infectious disease specialist, rehabilitation specialist, psychologist, and transplant specialist, as well as comprehensive laboratory and instrumental support: high-resolution chest CT, comprehensive assessment of pulmonary function, including spirometry, body plethysmography with measurement of the lung diffusion capacity, echocardiography with pulmonary artery blood pressure, electrocardiography, pulse oximetry, assessment of laboratory activity of SJIA, and tests reflecting the activity of the MAS.

## Figures and Tables

**Table 1:** The main features of patients with and without respiratory system involvement at the onset of the SJIA.

Parameters	Whole Group *n* = 200 (%)	SJIA without RSI, *n* = 150 (%)	SJIA with RSI, *n* = 50 (%)	*p*-Value
***Demography***				
Age of SJIA onset, years, Me (25%:75%) min-max	5.3 (3.0: 9.1) 0.6–17.4	6.0 (3.2: 9.1) 0.6–16.9	4.9 (2.4: 9.1) 0.8–17.4	0.312
Observation, years, Me (25%:75%) min-max	2.0 (1.0; 3.0) 1.0–17.0	2.0 (1.0; 4.0) 1.0–14.0	2.0 (1.0; 3.0) 1.0–17.0	0.162
Females, *n* (%)	118 (59.0)	88 (58.7)	30 (60.0)	0.868
***Clinical features***				
Rash, *n* (%)	153 (76.5)	110 (73.3)	43 (86.0)	0.067
Arthritis, *n* (%)	109 (54.5)	89 (59.3)	20 (40.0)	0.017
Arthralgia without arthritis, *n* (%)	20 (10.0)	14 (9.3)	6 (12.0)	0.586
Splenomegaly, *n* (%)	92 (46.0)	58 (38.7)	34 (68.0)	0.0003
Hepatomegaly, *n* (%)	131 (65.5)	92 (61.3)	39 (78.0)	0.032
Lymphadenopathy, *n* (%)	123 (61.5)	89 (59.3)	34 (68.0)	0.275
Coagulopathy, *n* (%)	21 (10.5)	10 (6.7)	11 (22.0)	0.002
Hemorrhage, *n* (%)	12 (6.0)	3 (2.0)	9 (18.0)	0.00004
Heart involvement, *n* (%)	48 (24.0)	21 (14.0)	27 (54.0)	0.0000001
CNS involvement, *n* (%)	29 (14.5)	17 (11.3)	12 (24.0)	0.028
Renal involvement, *n* (%)	21 (10.5)	9 (6.0)	12 (24.0)	0.003
Systemic score Me (25%:75%) min-max	2 (2; 3) 1–4	2 (2; 3) 1–4	3 (2; 4) 1–4	0.009
Active joints Me (25%:75%) min-max	3.0 (1.0:8.0) 0.0–64.0	4.0 (1.0:10.0) 0.0–64.0	2.0 (0.0:5.0) 0.0–23.0	0.040
***Laboratory parameters***				
Hemoglobin, g/L, Me (25%;75%) min-max	100 (89; 111) 55–142	10 3 (91;113) 55–142	90 (81; 106) 60–127	0.001
WBC × 10^9^/L, Me (25%;75%) min-max	15.1 (9.0; 22.8) 0.1–102.0	14.6 (9.4; 22.4) 2.2–101.0	16.9 (5.9; 26.8) 0.1–102.0	0.964
Platelets × 10^9^/L, Me (25%;75%) min-max	394 (237; 583) 0–1099	443 (305; 593) 43–1099	201 (87; 492) 0–1038	0.00006
ESR, mm/h, Me (25%;75%) min-max	45 (28; 60) 2–120	45 (30; 60) 2–120	42 (22; 64) 2–120	0.944
C-reactive protein, mg/L, Me (25%; 75%) min-max	69 (25;131) 0–423	62 (25; 118) 0–423	103 (33;154) 0.3–300	0.111
ALT, U/L, Me (25%;75%) min-max	33 (19; 78) 6–2558	29 (17; 59) 6–911	49 (27; 200) 6–2558	0.002
AST, U/L, Me (25%;75%) min-max	37 (25; 79) 0–4862	33 (24; 55) 0–695	94 (38; 244) 13–4862	0.0002
GGT, U/L, Me (25%;75%) min-max	37 (21; 98) 8–726	30 (20; 67) 8–726	86 (32; 199) 12–667	0.010
ALP, U/L, Me (25%;75%) min-max	207 (141; 359) 11–1096	220 (142; 357) 11–1096	176 (109; 417) 75–1064	0.908
LDH, U/L, Me (25%;75%) min-max	487 (276; 768) 36–4240	451 (257; 624) 36–2770	801 (317; 2007) 124–4240	0.0005
Ferritin, ng/mL, Me (25%;75%) min-max	533 (159; 2013) 16–111,832	383 (139; 1500) 16–73,241	1909 (748; 12,000) 31–111,832	0.000006
Triglycerides, Me (25%;75%) min-max	1.8 (1.0; 3.0) 0.5–108	1.3 (1.0; 2.4) 0.5–107	2.8 (2.0; 3.9) 0.8–1080	0.0001
Prothrombin, % Me (25%;75%) min-max	86 (73; 101) 15–140	89 (78; 103) 53–128	75 (63; 93) 15–140	0.007
Fibrinogen, g\L, Me (25%;75%) min-max	4.3 (2.0; 6.0) 0.7–101.0	5.0 (3.1; 6.0) 1.0–101.0	2.0 (1.0; 4.5) 0.7–10.0	0.00002
Albumin, g\L, Me (25%;75%) min-max	36.5 (26.0; 43.0) 18.0–58.0	39.0 (32.0; 43.0) 21.0–58.0	24.0 (23.0; 26.0) 18.0–39.0	0.000008
**MAS assessment**				
HScore points, Me (25%;75%) min-max	103 (72; 155) 23–299	91 (71; 130) 23–284	156 (110; 220) 33–299	0.0000001
HScore %, Me (25%;75%) min-max	2 (1; 32) 0–99	1 (1; 7) 0–99	32 (2; 96) 1–99	0.0000001
MAS-HLH2004-criteria [14] *n* (%)	22/189 (11.6)	5/144 (3.5)	17/45 (37.8)	0.0000001
MAS 2005-criteria [13], *n* (%)	28/189 (14.8)	10/144 (6.9)	18/45 (40.0)	0.0000001
MAS EULAR/ACR/PRINTO criteria 2016 [12], *n* (%)	33/189 (17.5)	13/144 (9.0)	20/45 (44.4)	0.0000001

**Abbreviations:** ALP—alkaline phosphatase; ALT—alanine transaminase; AST—aspartate aminotransferase; CNS—central nervous system involvement; ESR—erythrocyte sedimentation rate; GGT—γ-glutamyltransferase; HLH—hemophagocytic lymphohistiocytosis; HScore—hemophagocytic score; LDH—lactate dehydrogenase; MAS—macrophage activation syndrome; Me—median; RSI—respiratory system involvement; SJIA—systemic juvenile idiopathic arthritis; WBC—white blood cells.

**Table 2:** The predictors are associated with **respiratory system involvement** at the onset of the SJI.

Predictors	OR (95% CI)	Se	Sp	*p*-Value
ICU admission	13.7 (4.7; 39.8)	32.0	96.7	0.0000001
Rash	2.2 (0.9; 5.4)	86.0	26.7	0.067
No arthritis	2.2 (1.1; 4.2)	60.0	59.3	0.017
Splenomegalia	3.4 (1.7; 6.7)	68.0	61.3	0.0003
Hepatomegalia	2.3 (1.1; 4.7)	78.0	38.7	0.032
Coagulopathy	4.0 (1.6; 10.0)	22.0	93.3	0.002
Hemorrhage	10.8 (2.8; 41.6)	18.0	98.0	0.00004
Heart involvement	7.2 (3.5; 14.9)	54.0	86.0	0.0000001
Neural involvement	2.5 (1.1; 5.6)	24.0	88.7	0.028
Renal involvement	5.0 (1.9; 12.6)	24.0	94.0	0.0003
Bone marrow hemophagocytosis	2.1 (1.1; 4.1)	46.0	71.3	0.024
Correspondence to HLH2004 criteria [14]	16.8 (5.7; 49.2)	37.8	96.5	0.0000001
Correspondence to MAS2016 criteria [12]	8.9 (3.7; 21.5)	40.0	93.1	0.0000001
No cervical spine arthritis	3.6 (1.05; 12.5)	94.0	18.8	0.031
No elbow arthritis	2.5 (0.9; 6.7)	90.0	21.5	0.071
No wrist arthritis	2.1 (1.02; 4.2)	74.0	42.3	0.040
Treatment with IVIG	6.4 (3.1; 13.3)	48.0	87.3	0.0000001
Hemoglobin ≤ 91.0 g/L	4.0 (1.9; 8.2)	58.5	73.7	0.0001
Platelets ≤ 211.0 × 10^9^	8.5 (3.8; 18.7)	54.8	87.5	0.0000001
ALT > 39.0 IU/L	3.3 (1.6; 6.9)	65.9	63.0	0.001
AST > 90.0 IU/L	7.8 (3.5; 17.3)	51.2	88.1	0.0000001
GGT> 42.0 IU/L	5.3 (1.8; 15.6)	68.2	71.2	0.002
LDH > 796.0 IU/L	7.0 (2.9; 17.3)	51.6	86.8	0.000005
Ferritin > 918.0 ng/mL	5.4 (2.4; 12.2)	73.7	65.8	0.00002
Triglycerides > 2.0 mmol/L	9.0 (3.2; 25.0)	78.6	71.1	0.000004
Prothrombin ≤ 75.0%	5.0 (2.0; 12.8)	54.8	80.6	0.0004
Fibrinogen ≤ 2.1 g/L	11.2 (4.3; 28.9)	63.9	86.3	0.0000001
Albumin ≤ 26.0 g/L	45.2 (8.0; 256.7)	78.6	92.5	0.0000001
Systemic count > 3.0	26.0 (5.6; 120.3)	26.0	98.7	0.0000001
HScore > 130.0	6.4 (3.1; 13.4)	65.9	76.9	0.0000001
HScore, % > 7%	5.7 (2.8; 11.9)	63.6	76.6	0.000001
Active joints ≤ 4	2.2 (1.1; 4.4)	74.0	33.3	0.030

**Abbreviations:** ALT—alanine transaminase; AST—aspartate aminotransferase; HLH—hemophagocytic lymphohistiocytosis; HScore—hemophagocytic score; ICU—intensive care unit; IVIG—intravenous immunoglobulin; LDH lactate dehydrogenase; MAS—macrophage activation syndrome; OR—odds ratio; Se—sensitivity; Sp—specificity.

**Table 3:** The outcomes of sJIA patients depend on the respiratory system involvement at the onset.

Outcomes	SJIA without RSI, *n* = 150 (%)	SJIA with RSI, *n* = 50 (%)	*p*-Value
Patients with remission, *n* (%)	132 (88.0)	39 (78.0)	0.082
Patients discontinued corticosteroids, *n* (%)	123 (82.0)	42 (84.0)	0.747
Withdrawn of nbDMARD, *n* (%)	33 (22.0)	12 (24.0)	0.770
Patients, who had at least one major flare, *n* (%)	21 (14.0)	7 (14.0)	1.0
Patients, who received bDMARD, *n* (%)	100 (66.7)	39 (78)	0.132
Remission on the first bDMARD, *n* (%)	74/100 (74)	27/39 (69.2)	0.594
Patients, whom bDMARD switched, *n* (%)	26/100 (26.0)	12/39 (24.0)	0.571
Second biologic, *n* (%) Tocilizumab Canakinumab Rituximab Abatacept Etanercept Tofacitinib Adalimumab Anakinra Canakinumab + Tofacitinib	5/26 (19.2) 10/26 (38.4) 1/26 (3.9) 2/26 (7.7) 2/26 (7.7) 1/26 (3.9) 4/26 (15.4) 1/26 (3.8) 0/26 (0.0)	2/12 (16.7) 4/12 (33.4) 0/12 (0.0) 1/12 (8.3) 2/12 (16.7) 0/12 (0.0) 1/12 (8.3) 1/12 (8.3) 1/12 (8.3)	0.816
Remission on the second biologic, *n* (%)	17/26 (65.4)	6/12 (50.0)	0.367
Patients, whom bDMARD re-switched, *n* (%)	10/26 (38.5)	5/12 (41.7)	0.851
Remission on the final biologic agent, *n* (%)	9/10 (90.0)	5/5 (100)	0.465
Overall remission, *n* (%)	145 (96.7)	48 (96.0)	0.824
Overall remission on bDMARD, *n* (%)	100/100 (100)	38/39 (97.4)	0.109
Patients with reduced bDMARD, *n* (%)	42/100 (42.0)	18 (46.2)	0.657
Tapering bDMARD, *n* (%)	32/100 (32.0)	14/39 (35.9)	0.661
bDMARD discontinuation, *n* (%)	10/100 (10.0)	4/39 (10.3)	0.964
Flare during/after bDMARD tapering/discontinuation, *n* (%)	0/35 (0.0)	1/16 (6.3)	0.135
Targets for biologics			
Successful target	*n* = 100 (%)	*n* = 39 (%)	
Interleukin-6 blockers	75 (75.0)	28 (71.8)	0.751
Interleukin-1 blockers	16 (16.0)	7 (18.0)	
Tumor necrosis factor-a blockers	7 (7.0)	2 (5.1)	
Others	2 (2.0)	2 (5.1)	
Infusion reaction on tocilizumab, *n* (%)	2 (2.0)	3 (8.1)	0.106

**Abbreviations:** DMARD—disease modification anti-rheumatic drug; bDMARD—biologic DMARD; nbDMARD—non-biologic DMARD.

## Data Availability

The datasets generated during and/or analyzed during the current study are available from the corresponding author upon reasonable request.
